# Neoadjuvant chemotherapy followed by interval debulking surgery in patients with serous endometrial cancer with transperitoneal spread (stage IV): a new preferred treatment?

**DOI:** 10.1038/sj.bjc.6605157

**Published:** 2009-06-30

**Authors:** I Vandenput, B Van Calster, A Capoen, K Leunen, P Berteloot, P Neven, Ph Moerman, I Vergote, F Amant

**Affiliations:** 1 Leuven Cancer Institute, Gynecologic Oncology, UZ Gasthuisberg, Katholieke Universiteit Leuven, Leuven, Belgium; 2 Department of Electrical Engineering – SCD, Katholieke Universiteit Leuven, Leuven, Belgium; 3 Department of Pathology, UZ Gasthuisberg, Katholieke Universiteit Leuven, Leuven, Belgium

**Keywords:** stage IV endometrial cancer, transperitoneal spread, neoadjuvant chemotherapy, interval debulking surgery

## Abstract

**Background:**

To investigate the value of neoadjuvant chemotherapy (NACT), followed by interval debulking surgery (IDS), in endometrial cancer with transperitoneal spread (stage IV).

**Methods:**

Patients with endometrial cancer with transperitoneal spread, as determined by laparoscopy (±pleural effusion), were treated with NACT. Efficacy was determined according to the Response Evaluation Criteria in Solid Tumors, residual tumour at IDS and histopathological assessment of tumour regression.

**Results:**

A total of 30 patients (median age: 65 years; range:44–81 years) received 3–4 cycles of NACT (83% paclitaxel/carboplatin). Histopathological subtypes were as follows: serous (90%), clear cell (3%) and endometrioid (6%) carcinoma. Response according to RECIST was as follows: 2 (7%) complete remission, 20 (67%) partial remission, 6 (20%) stable disease and 2 (7%) progressive disease (PD). Patients with PD were not operated upon. A total of 24 patients (80%) had optimal cytoreduction (*R* ⩽1 cm), of whom 22 (92%) were without residual tumour. Four patients were considered inoperable and were excluded from further analysis. The median progression-free survival and overall survival times were 13 and 23 months, respectively.

Histopathological features of chemoresponse in both uterus and omentum were related to a better PFS (*P*=0.017, hazard ratio (HR) =0.785) and overall survival (*P*=0.014, HR=0.707). In particular, the absence of tumour infiltration and necrosis were associated with prognosis.

**Conclusion:**

The use of NACT resulted in a high rate (80%) of optimal IDS for the treatment of endometrial cancer with transperitoneal spread.

Endometrial cancer is the most frequent malignancy of the female genital tract in the Western world. The most common subtype is endometrioid endometrial cancer (EEC) (80%). Uterine papillary serous carcinoma (UPSC) accounts for 10% and is a highly aggressive variant that is more likely to spread intraperitoneally, similar to ovarian cancer (Amant *et al*, 2005). The incidence of surgical stage IV disease is ∼5–10%, with a 5-year overall survival (OS) ranging from 0–10% (Goff *et al*, 1994). Despite the poor outcome, few data exist regarding the optimal management of patients with stage IV endometrial cancer.

The amount of residual disease after surgery for advanced endometrial cancer has an impact on median survival and progression-free interval (PFI) (Goff *et al*, 1994; Chi *et al*, 1997; Bristow *et al*, 2001; Memarzadeh *et al*, 2002; Lambrou *et al*, 2004; Thomas *et al*, 2007). These data correspond to findings in ovarian cancer. Primary debulking is, however, associated with a considerable rate of postoperative complications: 34–53% in ovarian cancer (Morice *et al*, 2003; Hou *et al*, 2007) and 36–39% in endometrial cancer (Bristow *et al*, 2001; Memarzadeh *et al*, 2002). Moreover, taking into consideration the age and co-morbidities of endometrial cancer patients, there is a need for better treatment options.

Neoadjuvant chemotherapy (NACT) has been used in the treatment of advanced ovarian cancer as an alternative approach to conventional primary debulking surgery (Chambers *et al*, 1990; Vergote *et al*, 1998; Kayikçioglu *et al*, 2001; Morice *et al*, 2003).

A strategy of NACT enables to identify chemo-sensitive disease that is more likely to benefit from debulking surgery when compared with chemoresistant disease. Furthermore, resection of a reduced tumour burden permits less aggressive surgery and can improve the patients' quality of life by reduced morbidity and shorter operations, intensive care unit stays and overall hospitalisations. A disadvantage of this neoadjuvant approach was the potential for a worse survival when compared with primary debulking. In addition, when the neoadjuvant approach was introduced, some reluctance existed regarding post-chemotherapy complications at the time of major surgery.

Cytotoxic therapy leads to morphological and histopathological changes within tumour tissue and in involved stromal tissue. Successful treatment results in fibrosis and scarring of tumoral stroma and the surrounding tissue. Histopathological tumour regression has been established as the gold standard for the assessment of treatment response in several types of solid tumours (osteosarcomas, gastric, oesophageal and non-small-cell lung cancer) (Sassen *et al*, 2007). A histopathological assessment of tumour regression in ovarian cancer patients showed a correlation between the composite pathological tumour response score and prolonged progression-free survival (Le *et al*, 2006; Sassen *et al*, 2007).

In endometrial cancer, the experience of NACT, followed by interval debulking surgery (IDS), is anecdotal (Resnik and Taxy, 1996; Le *et al*, 1999; Price *et al*, 1999; Despierre *et al*, 2006). Given the advantages of this strategy, we prospectively investigated the value of NACT, followed by IDS, for stage IV endometrial cancer, and tested the hypothesis that the histopathological assessment of response is predictive for the outcome.

## Materials and methods

Between October 1999 and October 2007, patients who were diagnosed with stage IV endometrial cancer by laparoscopy at the UZ Gasthuisberg Leuven (Belgium) were enrolled in the study. Patients with ascites or peritoneal/omental disease diagnosed using a computed tomography (CT) scan were selected to undergo a laparoscopy and a curettage. The majority of patients had an endometrial evaluation showing a primary uterine source. However, two cases in the beginning of this study were treated as ovarian cancer, but a final histology at the time of IDS correlated with endometrial cancer.

The operative notes of the laparoscopy describe the presence of ascites, the number of metastases and the diameter of the largest metastases in the following organs: stomach, gallbladder, spleen, omentum, bladder, colon, rectum, small bowel, uterus, etc. Further, we describe peritoneal disease at the left and right diaphragm, the left and right paracolic gutter, pelvis and abdominal wall.

Eligibility criteria were patients with primary and measurable stage IV endometrial cancer based on transperitoneal spread as determined by laparoscopy with or without pleural effusion. Patients who were exposed to ineffective primary surgery resulting in suboptimal debulking were also included. In our institution, patients with lung or liver metastases are treated with chemotherapy only, unless a complete remission (CR) in these distant organs is visualised on CT imaging. These patients were excluded from the study.

All patients received 3–4 cycles of NACT before analysis. In case of stable disease (SD), partial remission (PR) or CR, IDS was performed. In case of progressive disease (PD), patients were excluded from the study. When the patient recovered from surgery, 2–3 additional cycles of chemotherapy were administered.

Efficacy was determined on three levels.

First, response was preoperatively evaluated on imaging studies using the Response Evaluation Criteria in Solid Tumors (RECIST) (Therasse *et al*, 2000). CA-125 levels were available, although the decision on response was based on RECIST only.

Second, at the time of IDS, response was assessed according to the macroscopic tumour load at the start and end of surgery. At the start of IDS, the surgeon made a subjective evaluation of good or no response to chemotherapy, with good response indicating that the tumour was replaced by an avascular and fibrotic lesion. Residual tumour was assessed at the end of surgery according to the following definitions: complete cytoreduction (no residual tumour), optimal cytoreduction (residual tumour <1 cm) and suboptimal cytoreduction (residual tumour of >1 cm).

Third, a histopathological analysis was carried out on tumour specimens that were obtained during IDS on the basis of the methodology described by Sassen *et al* (2007). Eight histopathological features of tumour regression were assessed in the uterus and omentum, namely, the presence of fibrosis, necrosis, inflammatory cell infiltrates, foamy macrophages, isolated psammoma bodies, giant cells of foreign-body type, giant tumour cells and pattern of tumour infiltration. For each feature, three grades were defined as follows: not or minimally present (0/1+), focal occurrence (2+) and widespread occurrence within the surgical specimen (3+). Fibrosis was classified as a regressive change only when extensive fibrosis was associated with little or no residual tumour.

The pattern and extent of tumour infiltration were classified as follows: macroscopic large confluent tumour mass (1+), multiple small tumour foci (2+) and scattered solitary tumour cells or a complete absence of residual tumour (3+).

An ‘overall tumour regression score’ was derived by adding the extent of regressive changes (0/1+=1; 2+=2; 3+=3) for all eight histopathological features in both organs. This overall tumour regression score can vary between 16 and 48, and was linked to progression-free survival (PFS) and OS using the Cox proportional hazards regression model. This allowed us to assess whether these regressive changes were an indicator of treatment response.

We controlled for the CA-125 level and response on CT by adding these as covariates. The limited sample size and number of events prohibited the investigation of more covariates (Vittinghoff and McCulloch, 2007).

Results were summarised by hazard ratio (HR), together with 95% profile likelihood confidence intervals (CIs). HR estimates the change in hazard of recurrence or death for a unit increase in the overall tumour regression score. The proportional hazards assumption was checked.

## Results

A total of 30 patients were enrolled in the study and their characteristics are shown in Table 1. The number of treatment adjustments due to grade 3–4 bone marrow toxicity is summarised in Table 2.

### Preoperative response after NACT

Response evaluation according to the RECIST criteria was as follows: CR in 2 (7%) patients, PR in 20 (67%) patients and SD in 6 (20%) patients. The response rate was 74%. A total of 2 patients (7%) showed progression.

### Response at IDS

During surgery, 4 (13%) patients were considered inoperable for the following reasons: invasion in bladder and rectum (*n*=1), involvement of superior mesenteric artery/porta hepatis (*n*=2) and extensive retroperitoneal disease (*n*=1). A total of 24 patients underwent IDS. According to the macroscopic tumour load, 20 patients (83%) showed clear signs of chemosensitivity, 2 (8%) had no obvious signs and data were not available for 2 (8%) patients. Surgical procedures were as follows: total abdominal hysterectomy with bilateral salpingo-öphorectomy and omentectomy (*n*=24), pelvic+paraaortic lymphadenectomy (*n*=12), douglasectomy (*n*=9), stripping of the diaphragm (*n*=2) and coagulation of the diaphragm (*n*=7). No splenectomy or bowel resections were performed.

A total of 22 out of 24 patients (92%) had complete cytoreduction (no residual tumour) and 2 (8%) had optimal cytoreduction (multiple lesions of <1 and 0.5 cm in each patient).

The pathology report of uterine specimens showed mainly a poorly differentiated serous papillary carcinoma (*n*=18), a well-differentiated endometrioid carcinoma (*n*=1) and only endometrial intraepithelial carcinoma (EIC) (*n*=3). In two cases, no residual tumour was found in the endometrium/myometrium. Myometrial invasion to the outer half was reported in 10 cases, cervical stromal invasion in 4 cases and lymph vessel space involvement in 8 cases. Of the 12 patients who underwent a lymphadenectomy, 6 (50%) showed node involvement.

**Figure 1:**
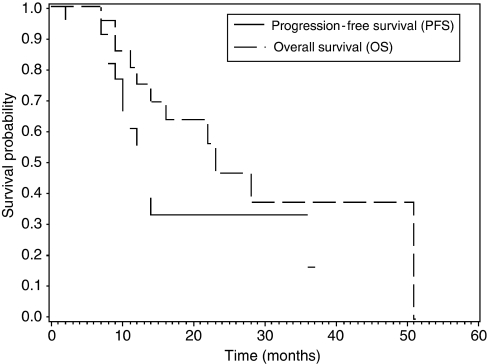
Kaplan–Meier curves for PFS and OS.

The median PFS time was 13 months, indicating that 50% of patients experienced disease progression within 13 months (Figure 1). The median OS time was 23 months (Figure 1). All patients of the group who were progressive or inoperable (*n*=6) died of disease. Median OS was 12 months (range: 5–24) for this group.

A total of 3 (13%) patients showed minor postoperative complications (urinary tract infection and prolonged time to bowel movement) and 1 (4%) patient needed a re-laparotomy because of internal bleeding.

### Histopathological response

A total of six patients were excluded because of PD or inoperability. A total of 24 patients received 2–5 cycles of postoperative chemotherapy, shown in Table 2.

**Figure 2:**
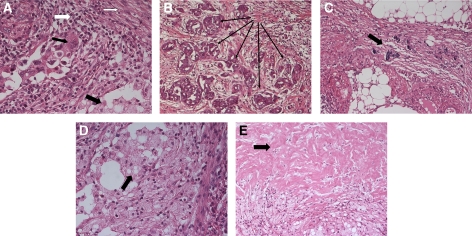
(**A** and **B**) Representative slides of good and poor histopathological response on chemotherapy in the omentum. (**A**) Good chemotherapy response (H&E × 40) is based on tumour inflammation (large white arrow), fibrosis (small white arrow), foamy macrophages (large black arrow) and tumour infiltration (small black arrow). (**B**) Poor chemotherapy response (H&E × 40) is based on the absence of regression criteria and multifocal tumour infiltration (black arrow). (**C**–**E**) Histopathological features of tumour regression (indicated by arrow) in endometrial cancer. **C** (H&E × 40): psammoma bodies; **D** (H&E × 40): foamy macrophages; **E** (H&E × 40): necrosis.

The results for the eight assessed features of histopathological tumour regression in the uterus and omentum are shown in Table 3 and illustrated in Figure 2.

**Figure 3:**
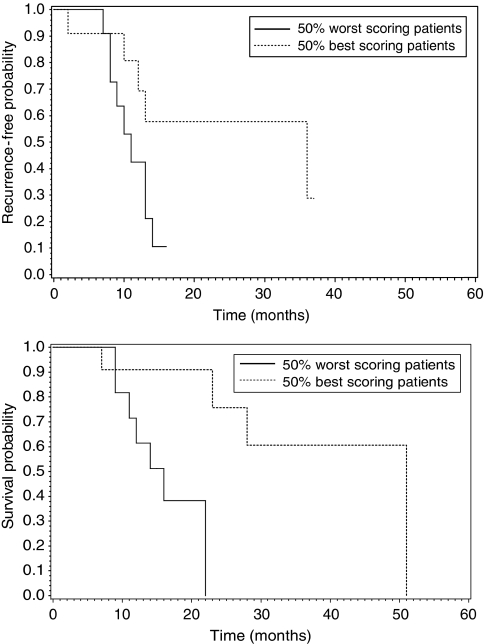
Correlation of all histopathological features with recurrence and overall survival.

Using Cox regression, we observed that the overall extent of tumour regression was related to a better PFS (HR=0.785, 95% CI 0.599–0.964) and a better OS (HR=0.707, 95% CI 0.482–0.943). These effects are visualised by Kaplan–Meier curves for the best and worst scoring patients on the basis of a median split (Figure 3).

Statistically controlling for CA-125 and response on CT did not change the results for PFS (HR=0.733, 95% CI 0.539–0.934) or OS (HR=0.692, 95% CI 0.479–0.923).

The features ‘fibrosis’, ‘necrosis’ and grade of ‘tumour infiltration’ were more often present than were other regressive changes (Table 3), and were separately analysed to tentatively investigate which features contributed most to the effect of the overall tumour regression score. It was suggested that less tumour infiltration and more necrosis were related to better PFS (infiltration: HR=0.721, 95% CI 0.475–1.081; necrosis: HR=0.341, 95% CI 0.087–0.878) and better OS (infiltration: HR=0.717, 95% CI 0.445–1.140; necrosis: HR=0.215, 95% CI 0.036–0.682), in contrast to fibrosis.

## Discussion

The clinical decision making for advanced stage endometrial cancer should preferably take morbidity attributed to primary debulking surgery into consideration. Endometrial cancer also frequently affects old and fragile women. Balancing between chemotherapy (without surgery) and aggressive cytoreductive surgery, when sensitivity to chemotherapy is not known, the use of NACT followed by IDS might be a valuable option. This study showed that this strategy is a valuable alternative as we were able to identify chemoresistant disease (and hence poor candidates for any surgery) and, given the high rate of complete cytoreduction, a low complication rate. These results confirm earlier case reports on this subject (Resnik and Taxy, 1996; Le *et al*, 1999; Price *et al*, 1999). It appears from Table 3 that all five previously published cases had CR or PR based on imaging studies and all had no residual disease after IDS.

The response to chemotherapy as determined by a histopathological assessment of the tumour is identified as a new prognostic marker in endometrial cancer. In patients with ovarian cancer undergoing IDS, Le *et al* (2006, 2007) assessed four features of tumour necrosis (necrosis, fibrosis, macrophage infiltration and tumour-induced inflammation). They concluded that a higher composite pathological tumour response score was significantly correlated with a prolonged progression-free survival (*P*=0.0016) and OS (*P*=0.017). In contrast, Sassen *et al* (2007) could not confirm this correlation between histopathological features and OS. Residual tumour size was the only criterion significantly correlated with treatment response and OS. In the absence of any data on this in endometrial cancer, we used the same criteria as that used for ovarian cancer (Sassen *et al*, 2007). In this study, a histopathological score indicative of chemosensitivity was significantly correlated with recurrence and OS. Especially, tumour infiltration and necrosis were related to better PFS and OS.

In current practice, patients with stage IV endometrial cancer receive either no surgery (systemic treatment only) or primary debulking surgery. An overview of studies exploring the latter is presented in Table 4. From this table, it seems that PFS and OS depend on the amount of residual disease and that patients without residual tumour have a better outcome. In addition, 28–48% of patients were unable to have optimal cytoreduction and 18–38% were considered inoperable. In contrast, this study revealed 24 patients (80%) who underwent optimal cytoreduction (*R* ⩽1 cm), of whom 22 (92%) were with no residual tumour. Only 4 (14%) patients were considered inoperable. The median PFS of 13 months corresponds to that of previous reports (Memarzadeh *et al*, 2002; Thomas *et al*, 2007).

In addition, the data on postoperative complications were better in this analysis. In studies depicted in Table 4, minor postoperative complications (wound infection, prolonged time to bowel movement, urinary tract infection, pneumonia and deep venous thrombosis) after primary debulking occurred in 36–39% of the cases. Major life-threatening complications (small bowel obstruction, myocardial infarction and pulmonary embolism) occurred in 13% of cases (Bristow *et al*, 2001; Memarzadeh *et al*, 2002). These data contrast to the current analysis in which minor and major postoperative complications were observed in 13 and 4% of cases, respectively.

Thus, comparing the data on efficacy and morbidity from studies depicted in Table 4, with the current findings, it seems that the investigated strategy results in a higher chance for complete surgical resection with less postoperative morbidity.

The value of IDS has been a focus of interest for a long time in the advanced stage of ovarian cancer. Numerous retrospective studies have documented the potential benefit of IDS in ovarian cancer. Recently, the results of a randomised trial of EORTC-GCG/NCIC-CTG comparing primary debulking with IDS after NACT in stage IIIc–IV ovarian, fallopian tube and peritoneal cancer were presented by I Vergote *et al* (Vergote *et al*, 2008). The data showed a similar OS and PFS. Vergote *et al* (Vergote *et al*, 2008) concluded that, in very advanced stage IIIc and IV ovarian carcinoma, as included in their study, NACT could be considered as an alternative for primary debulking surgery because of the lower morbidity of IDS compared with primary debulking. A majority of our patients had serous-type endometrial cancer stage IV based on transperitoneal spread, resembling the EORTC-GCG/NCIC-CTG study population. Given these similarities and given our current findings, we believe that the data obtained in stage IIIc ovarian cancer might be extrapolated to endometrial cancer with transperitoneal spread (stage IV). This is an important notion as a large comparative study in endometrial cancer with transperitoneal spread is unlikely to be feasible.

In conclusion, the degree of tumour regression after NACT for advanced stage endometrial cancer was identified as a new prognostic marker. The use of NACT for stage IV endometrial cancer resulted in an 80% optimal cytoreduction in patients, wherein an attempt for complete resection was made with a low postoperative morbidity rate. In combination with the most recent data in ovarian cancer with transperitoneal spread, the current data suggest that NACT followed by IDS is a valuable option for endometrial cancer with transperitoneal spread.

## Figures and Tables

**Table 1:** Patient characteristics

	***n* (%)**
Total of patients	30
	
*Age (years)*	
Median (range)	65 (44–81)
	
*Menopausal status*	
Pre	1 (3)
Peri	1 (3)
Post	28 (93)
	
*Histological subtype*	
Serous	27 (90)
Clear cell	1 (3)
Endometrioid	
Grade 1	1 (3)
Grade 2	1 (3)
	
*Type of NACT*	
Paclitaxel/carboplatin	25 (83)
Doxorubicin/cisplatin	3 (10)
Epirubicin/carboplatin	1 (3)
Carboplatin	1 (3)
	
*Number of cycles before IDS*	
3	27 (90)
4	3 (10)

*n*=number of patients; NACT=neoadjuvant chemotherapy; IDS= interval debulking surgery.

**Table 2:** Overview of treatment adjustment because of haematological toxicity in neo-adjuvant period and postoperative period

	**Neo-adjuvant chemotherapy**		**Post-operative chemotherapy**	
	***n* patients (%)**	***n* cycles (%)**	***n* patients (%)**	***n* cycles (%)**
Total	30 (100)	93 (100)	24 (100)	72 (100)
Dose delay	12 (40)	14 (15)	8 (33)	8 (11)
Dose reduction	2 (7)	5 (5)	5 (21)	13 (18)
Switch to other types of chemotherapy	0	0	2 (8)	4 (6)

*n*=number.

**Table 3:** Results of scoring all histopathological features (*n*=8) of tumour regression in uterus and omentum

**Histopathological features**	**Uterus *n* (%)**	**Omentum *n* (%)**
*Necrosis*		
0/1+	22 (92)	19 (79)
2+	1 (4)	3 (13)
3+	1 (4)	2 (8)
		
*Fibrosis*		
0/1+	15 (63)	6 (25)
2+	7 (29)	6 (25)
3+	2 (8)	12 (50)
		
*Inflammation*		
0/1+	20 (83)	20 (83)
2+	2 (8)	1 (4)
3+	2 (8)	3 (13)
		
*Psammoma bodies*		
0/1+	20 (83)	15 (63)
2+	4 (17)	5 (21)
3+	0	4 (17)
		
*Foamy macrophages*		
0/1+	22 (92)	21 (88)
2+	1 (4)	0
3+	1 (4)	3 (13)
		
*Foreign-body giant cells*		
0/1+	24 (100)	22 (92)
2+	0	2 (8)
3+	0	0
		
*Giant tumour cells*		
0/1+	21 (88)	20 (83)
2+	3 (13)	3 (13)
3+	0	1 (4)
		
*Tumour infiltration*		
1+	12 (50)	12 (50)
2+	3 (13)	3 (13)
3+	9 (38)	9 (38)

*n*=number of patients.

**Table 4:** Overview of studies investigating the role of cytoreduction in patients with advanced stage endometrial cancer

**Study**	***n***	**Stage**	**Type**	**Treatment**	**Residual disease**	**%**	**PFS (mts)**	**OS (mts)**
Chi *et al* (1997)	55	IV	EEC+UPSC	Primary surgery	⩽2 cm	44		31
					>2 cm	38		12
					Inoperable	18		3
Goff *et al* (1994)	47	IV	EEC+UPSC	Primary surgery	No gross bulky disease	62		18
					Inoperable	38		8
Memarzadeh *et al* (2002)	35	IIIc+IV	UPSC	Primary surgery	0	57	22	40
					Macroscopic	43	8	10
Bristow *et al* (2001) a	31	IV	UPSC	Primary surgery	⩽1 cm	52		26
					>1 cm	48		10
Lambrou *et al* (2004) b	58	IIIc+IV	EEC	Primary surgery	⩽2 cm	72		18
					>2 cm	28		7
Thomas *et al* (2007) a	70	IIIc+IV	UPSC	Primary surgery	0	37	9	51
					⩽1 cm	60	6	14
Resnik and Taxy (1996)	1	IV	UPSC	NACT+IDS	0	100	7	
Price *et al* (1999)	3	IIIc+IV	UPSC	NACT+IDS	0	100	11	17
Le *et al* (1999)	1	IV	UPSC	NACT+IDS	0	100	6	
Current study^a^	30	IV	EEC+UPSC	NACT+IDS	⩽1 cm	80	13	23
					Inoperable	13		12

*n*=number of patients of total group; %=percentage of patients of the total group; PFS=progression-free survival; OS=overall survival; mts=months; EEC=endometrioid endometrial carcinoma; UPSC=uterine papillary serous carcinoma; NACT=neoadjuvant chemotherapy; IDS=interval debulking surgery.

a Optimal cytoreduction defined as ⩽1 cm.

b Optimal cytoreduction defined as ⩽2 cm.
